# Simulation and Analysis of Coherent Wind Lidar Based on Range Resolution

**DOI:** 10.3390/s25082344

**Published:** 2025-04-08

**Authors:** Jiaxin Chen, Hong Li, Weiwei Zhan, Yunkai Dong, Liheng Wu, Wenbo Wang

**Affiliations:** National Institute of Natural Hazards, Beijing 100085, China; cjx1090402764@163.com (J.C.); hank116@126.com (W.Z.); dongyunkai2012@163.com (Y.D.); smiling-lily8013@163.com (L.W.); wwb_wwb@126.com (W.W.)

**Keywords:** coherent wind lidar, range resolution, short-pulse method, simulation model

## Abstract

The wind field, a critical atmospheric parameter, significantly influences climate, weather forecasting, aviation safety, and wind energy applications. The precise observation of wind fields is essential for improving weather predictions, studying climate change, ensuring aviation safety, and optimizing wind energy systems. Among the various wind field detection methods, coherent wind lidar technology stands out due to its superior detection range, accuracy, and robustness. However, the high-range resolution required for applications such as aircraft takeoff and landing or wind turbine region monitoring presents unique challenges in wind detection. To address the aforementioned challenges, this study established a modular coherent Doppler wind lidar simulation system. Unlike traditional single-module simulation approaches, this system achieves multi-parameter coupling analysis of laser emission under pulse modulation, atmospheric transmission, and wind speed inversion through integrated hardware-transmission-processing collaborative modeling. Subsequently, by adjusting key parameters of the system model, an in-depth analysis of wind speed inversion within a 1.2 km detection range was conducted, investigating the dual impacts of reducing pulse duration on both range resolution and wind speed measurement accuracy. Furthermore, a Mach–Zehnder modulator module was implemented in the radar hardware section to generate odd–even pulse pairs, while a differential correlation algorithm was introduced in the data processing module to enhance range resolution. Ultimately, wind speed measurements with a 4.5 m range resolution along the laser emission direction were achieved in simulations. Comparative analysis shows that pulse modulation techniques effectively reduce wind speed measurement errors caused by short-pulse methods, offering a reliable framework for practical wind field measurements.

## 1. Introduction

The wind field is a critical atmospheric parameter that influences climate, weather forecasting [1], aviation safety [2,3], and wind energy utilization [4,5]. The accurate observation of wind fields is essential for reducing economic losses from severe weather, advancing climate change research [6], ensuring flight safety, and optimizing renewable energy generation. The World Meteorological Organization highlights wind field measurements as a vital dataset for weather prediction. In climate studies, the accurate analysis of atmospheric variables, including wind field distribution, atmospheric transport, and material fluxes, is indispensable. However, current wind field measurement techniques face limitations in accuracy and range resolution, particularly in applications requiring a detailed analysis of small-scale vortex structures, such as those found in wind tunnels and aircraft wake vortices. For instance, existing commercial all-fiber coherent Doppler wind lidars (CDWLs) achieve a minimum range resolution of 15 m [7], which falls short of the sub-10 m resolution needed for these applications [8].

In aviation safety, the complexity of low-altitude atmospheric wind fields poses significant risks during aircraft take-off and landing. Data from the International Air Transport Association (IATA) reveal that 53% of global commercial aviation accidents between 2005 and 2023 occurred during the landing phase. When including the takeoff phase (accounting for 8.5%), the combined proportion of accidents during these critical flight stages reached 61.5% [9]. The high-precision monitoring of atmospheric wind fields in these areas is therefore essential to mitigate flight risks. Similarly, wind energy, a rapidly growing renewable energy source, requires detailed wind field measurements in turbine regions to enhance power generation efficiency. Consequently, the development of high-range resolution wind field measurement technologies has significant practical value across multiple fields.

The CDWL utilizes the Doppler effect to gather information about the wind field. This tool is known for its real-time performance and robust detection capability, finding extensive applications in meteorological research and aviation safety [10]. With the development of fiber laser theory and fiber communication technology, the 1.5–1.6 μm band all-fiber coherent wind lidar system has gradually matured. In the past decade, the 1.5–1.6 μm band all-fiber wind measurement lidar system has developed towards long-distance, high-efficiency, and miniaturization, and the measurement mode has also shifted from radial wind speed measurement to three-dimensional wind field detection. In recent years, the demand for aviation safety and the development of atmospheric turbulence research have raised higher range resolution requirements for wind field detection [11]. Researchers have gradually shifted their focus from the lidar detection range to high-range resolution.

The range resolution of pulsed CDWLs is determined by the width of the emitted laser pulse. The simplest way to improve range resolution is to shorten the pulse width.

In 2015, NASA developed the Windimager, a fully fiber-optic CDWL system capable of flexible pulse width control. This system achieved a minimum range resolution of 15 m, a wind measurement range of up to 10 km, and wind speed measurements of up to 120 m/s with an accuracy of ±0.2 m/s [7]. In 2020, Ocean University of China introduced Wind3D 10K, a commercialized long-range scanning wind lidar system. This system, based on a 1.55 μm all-fiber CDWL, demonstrated stable performance in diverse environments, with a maximum measurement range of 15 km and a minimum radial resolution of 15 m [12].

While shortening the pulse width enhances range resolution, it also increases the spectral width of backscatter signals, reducing wind speed measurement accuracy. Furthermore, at limited peak power, the weakened backscatter signal reduces the signal-to-noise ratio, diminishing the detection range.

To address these challenges, the University of Science and Technology of China (USTC) developed innovative pulse coding techniques to improve CDWL range resolution without compromising accuracy. In 2019, researchers employing Golay-coded pulse intensity modulation techniques can mitigate signal aliasing artifacts and enhance pulse repetition frequency (PRF), thereby compensating for the reduced measurement accuracy induced by narrow pulse widths under fixed single-pulse energy conditions. This method achieved a range resolution of 6 m and a maximum detection range of 552 m, with a wind profile acquisition time of 2 s [13]. Subsequently, in 2021, the USTC introduced a high-resolution CDWL system employing Pseudo-Random Phase Coding (PRPC). This technique achieved radial wind field measurements with a range resolution of 4.5 m and 1 s temporal resolution over a range of 800 m under a peak laser power of 250 W [14]. The team also proposed the Differential Correlation Pair (DCP) [15] and Joint Phase Shift Keying Differential Correlation Pair (PSK-DCP) techniques [16], achieving continuous radial wind field measurements with a 3 m range resolution and a 0.1 s temporal resolution under a peak power of 400 W.

To address the challenges of structural complexity and limited range resolution in CDWL systems, this study established an end-to-end system simulation model that dynamically simulates the entire process of laser emission, atmospheric transmission, and wind field inversion, providing theoretical support for breaking through traditional hardware design limitations. Based on this model, the impacts of key technologies, such as pulse modulation and non-coherent accumulation processing on range resolution, can be quantitatively analyzed, thereby enabling the proposal of a coordinated system parameter optimization strategy.

This study established an end-to-end CDWL system simulation model using numerical simulation tools. By adjusting key parameters in the simulation, a systematic comparison was conducted on the enhancement effects of two technical approaches for improving wind measurement range resolution: the first employs an acousto-optic modulator (AOM) to achieve dynamic pulse width compression, while the second implements an odd–even pulse coding scheme based on a Mach–Zehnder modulator (MZM) in the model and introduces a differential cross-correlation algorithm during the signal demodulation stage to enhance range resolution. Through the comparative validation of wind speed retrieval using both AOM pulse compression and MZM differential coding modulation modes within the full-link lidar simulation model, this work reveals the enhancement mechanism of time–frequency domain joint optimization on range resolution, providing a model foundation and theoretical basis for improving the performance of wind measurement systems.

## 2. Analysis of the Theoretical Simulation System of Coherent Wind Lidar Performance

**Figure 1:**
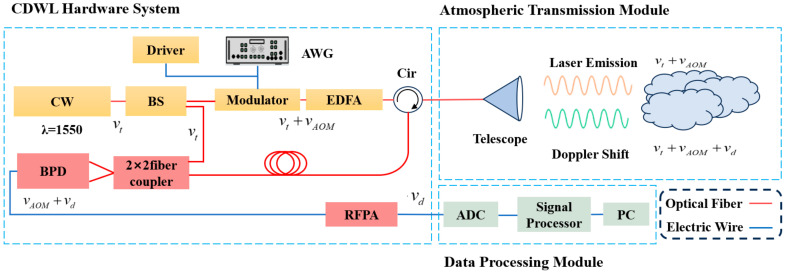
Schematic diagram of the coherent wind lidar system.

The principle of the traditional CDWL system is illustrated in Figure 1. In the laser emission system, a narrow-linewidth continuous wave laser (CW) generates a seed laser with a frequency of $v_{t}$, which is divided into two beams using a 1 × 2 beam splitter (BS). One beam serves as the local oscillator light (LO), which beats with the echo light signal; the other beam is used as the detection light. After modulation and frequency shifting by the optical modulator, a single-frequency pulse laser with a frequency of $v_{t}+v_{AOM}$ is generated. This laser is then amplified by the pulse laser amplifier and emitted into the atmosphere through a telescope via a circulator.

The emitted laser pulse interacts with aerosol particles in the atmosphere. Assuming the Doppler frequency shift $v_{d}$ is induced by the wind field, the frequency of the backscattered signal becomes $v_{t}+v_{AOM}+v_{d}$. The atmospheric laser echo signal is received by the optical telescope and, after passing through a 2 × 2 fiber coupler, is fed into the balanced detector for beating. The beat signal is converted into an intermediate-frequency electrical signal with a frequency $v_{AOM}+v_{d}$ by the balanced detector. This signal is then processed by the data acquisition and processing module, which performs filtering, amplification, analog-to-digital conversion [17], range gate division, Fourier transform, accumulation, and denoising. Finally, the wind speed is derived from the processed data.

### 2.1. The Establishment of the Simulation Model Based on the Laser Lidar System

**Figure 2:**
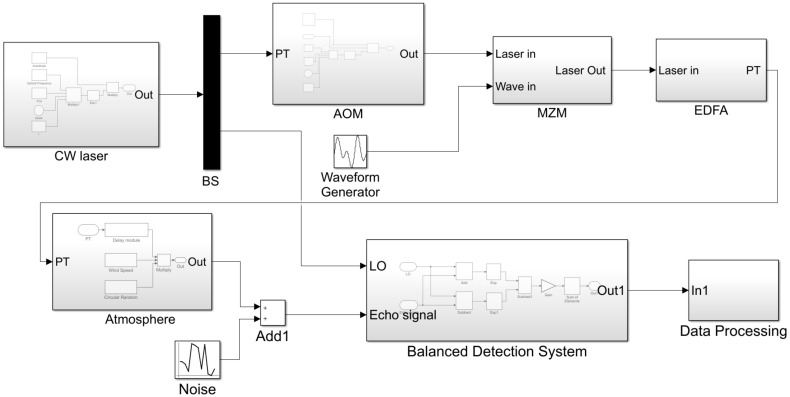
Coherent wind lidar system simulation block diagram.

Based on the aforementioned CDWL principles, the system simulation model was established, as shown in Figure 2. The CDWL hardware system includes a CW laser, an optical modulator, a balanced detection system, etc., primarily simulating the propagation process of laser emission within the radar system. The atmospheric system simulates the interaction between emitted laser pulses and atmospheric aerosol particles, as well as the subsequent collection of backscattered signals by the optical telescope. The data processing module incorporates algorithms such as rang gate division, power spectrum estimation, accumulative denoising, and wind speed retrieval.

**Figure 3:**
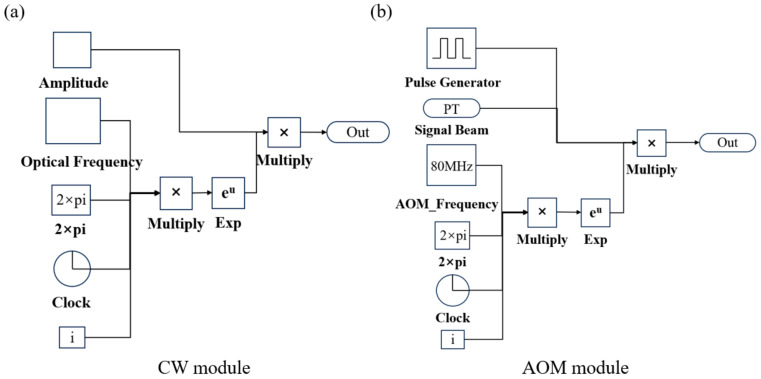
CW and AOM module simulation model diagram. (**a**) CW module; (**b**) AOM module.

The optical field emitted by a CW laser is typically expressed as $E(t)=A(t)\exp(j2\pi f_{c}t+j\varphi_{0})$, where A(t) is the amplitude envelope of the laser, $f_{c}$ is the carrier frequency, and $\varphi_{0}$ is the initial phase of the optical field. As indicated by the formula, the CW laser module is primarily composed of a multiplication module that supports complex number operations. The CW simulation model is illustrated in Figure 3a. The BS utilizes a Demux module to divide the signal into two paths, with each output path incorporating a Gain module to adjust the energy ratio after splitting.

The AOM was primarily constructed using a multiplication module. The Pulse_Generator converts the initial CW laser into pulsed laser emission at a specified frequency. In the frequency modulation section, a timer (Clock) introduces the frequency shift (f_AOM) into this module, and its input signal formula can be expressed as $\exp[i2\pi v_{AOM}t]$, with the AOM simulation model illustrated in Figure 3b. The MZM module will be detailed in the following section. The Erbium-Doped Fiber Amplifier (EDFA) employs a Gain module to achieve the desired output optical power for effective detection.

Based on the Back-Propagated Local Oscillator (BPLO) [18] computational method, the specific expression for the laser echo signal in the m-th stratified layer is derived as follows:

(1) $$\begin{array}{l} S(t)=2{\left(P_{\mathrm{LO}}\right)}^{1/2}\exp(i2\pi v_{AOM}t)\sum_{m}{\left[P_{T}\left(t-\frac{2R_{m}}{c}\right)\right]}^{1/2}\\ \times \exp\left[i\varphi \left(t-\frac{2R_{m}}{c}\right)\right]{\tilde{K}}_{m}\exp\left(-i2kv_{m}t\right) \end{array}$$

where $P_{LO}$ is the laser local oscillator power, $v_{AOM}$ is the AOM frequency shift, $P_{T}$ is the pulsed laser power, $R_{m}$ is the distance between the center of each layer and the receiving plane, m is the atmospheric layer number, φ is the phase function of the emitted laser, k is the wave number, and $v_{m}$ is the radial wind speed along the beam propagation axis. ${\tilde{K}}_{m}$ is a circular random variable.

In the simulation, the atmospheric system is established based on the stratified atmospheric model, which primarily consists of modules such as the delay module, wind speed, circular random, and product. The modulated PT serves as the input signal to the atmospheric system and enters the delay module. This module simulates the time-domain delay of the transmitted laser in the stratified atmospheric model, where different delay times are set to describe the echo signals reflected from different layers of the pulsed laser. The total echo signal is obtained by superimposing the signals from each layer. The delay term of the pulsed laser in the corresponding formula for the delay module is ${\left[P_{T}(t-\frac{2R_{m}}{c})\right]}^{1/2}\times \exp[i\varphi (t-\frac{2R_{m}}{c})]$. The relevant parameters for wind speed and circular random are imported via calculations from the From Workspace module. The wind speed module is utilized to set the radial wind speed $V_{m}$ throughout the entire detection range in the simulation, corresponding to the Doppler frequency shift term $\exp\left(-i2kv_{m}t\right)$ caused by the radial wind speed in the formula. The circular random variable, composed of atmospheric parameters, range, and telescope parameters, can be expressed as follows:

(2) $$\langle \left|K_{m}\right|\rangle =T{\left(R_{m}\right)}^{2}\beta (R_{m})\frac{A_{r}}{R_{m}^{2}}\eta_{m}\Delta R$$

$T(R_{m})$ denotes the atmospheric one-way transmittance, $\beta (R_{m})$ is the aerosol backscattering coefficient, $A_{r}=\pi D^{2}/4$ is the telescope receiving area, D is the effective telescope aperture, and $\eta_{m}$ is the system efficiency. ΔR denotes the minimum stratified distance of the atmospheric layers. The aforementioned parameters are also computed and imported via the from workspace module.

**Figure 4:**
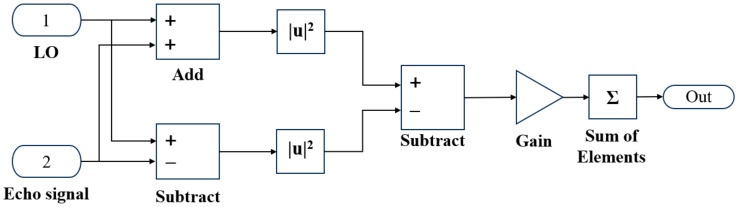
Balanced detection system simulation model diagram.

The simulation model of the balanced detection system is illustrated in Figure 4. Here, In1 represents the local oscillator (LO) optical signal, In2 corresponds to the atmospheric echo signal, and noise simulates environmental atmospheric noise added to the echo signal. The echo signal and LO signal pass through a 2 × 2 fiber coupler to generate interference signals, which are then fed into the balanced photodetector. The balanced photodetector performs differential processing on the two interference signals with opposite phases. The photocurrent signal is first converted to a voltage signal through a transimpedance amplifier (TIA), implemented as a gain module in the simulation. Subsequently, a summation module performs non-coherent accumulation of the backscattered echoes from each atmospheric layer, integrated over the pulse width duration.

The data acquisition and processing section primarily exports the detected voltage outputs to the workspace via the to workspace module for subsequent processing and includes algorithms such as range-gate division, power spectrum estimation, accumulative denoising, and wind speed retrieval.

### 2.2. Simulation Analysis of Laser Lidar Echo Signal Under Short Pulse Conditions

The range resolution ΔR of CDWL wind measurement is determined by the pulse width ΔT of the emitted laser pulse:

(3) $$\Delta R=\frac{\Delta Tc}{2}$$

where ΔT is the laser pulse width and c is the speed of light. The spectral width of the backscattering signal is also influenced by the emitted laser pulse width:

(4) $$\Delta w=\frac{\sqrt{ln2}}{\sqrt{2}\pi \Delta T}$$

From these equations, it is evident that the spectral width of the received echo signal is inversely proportional to the emitted laser pulse width. Therefore, to improve range resolution, the pulse width needs to be reduced. However, reducing the pulse width introduces the following challenges: 1. Due to hardware limitations, the peak power of the emitted laser pulse has an upper limit. Narrowing the pulse width reduces the energy of a single pulse, thereby lowering the signal-to-noise ratio. 2. Spectrum broadening increases the wind speed measurement error. As these equations suggest, while reducing the pulse width can improve the range resolution of wind measurements to some extent, it inevitably impacts wind speed measurement accuracy. Therefore, this section focuses on investigating the range resolution of the CDWL under short-pulse conditions through the established simulation model.

In this simulation, the wind speed module was configured to operate in the constant wind speed mode (0 m/s), while the noise module was set with a mean of 0 and variance of 10^−12^ W, representing the average noise power. Initially, the lidar simulation system was parameterized according to the specifications listed in Table 1, followed by wind speed retrieval within the 0–1.2 km range.

**Figure 5:**
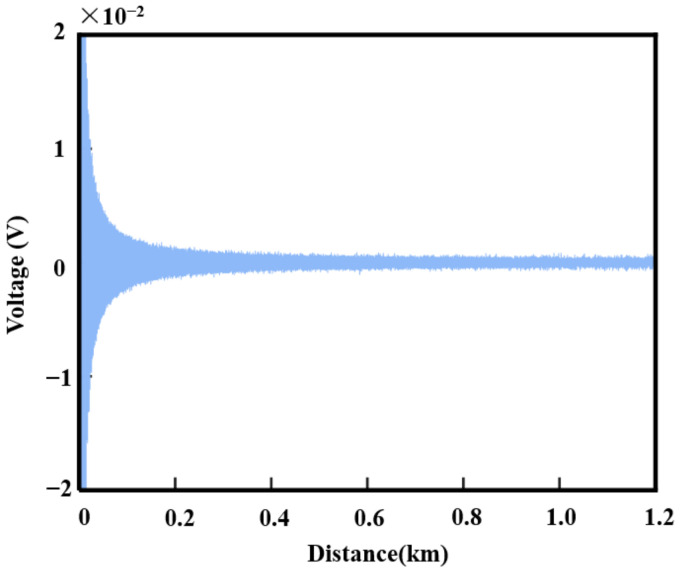
Balanced detector output voltage signal.

To observe how the balance detector output voltage varies with the detection distance, the pulse width was set to 40 ns, and the pulse repetition frequency was set to 10 kHz. The peak power of pulses of different widths was uniformly amplified to 100 W and emitted into the atmosphere. With a telescope aperture of 30 mm, the wind speed was set to 0 m/s. The balance detector output voltage signals are shown in Figure 5.

**Figure 6:**
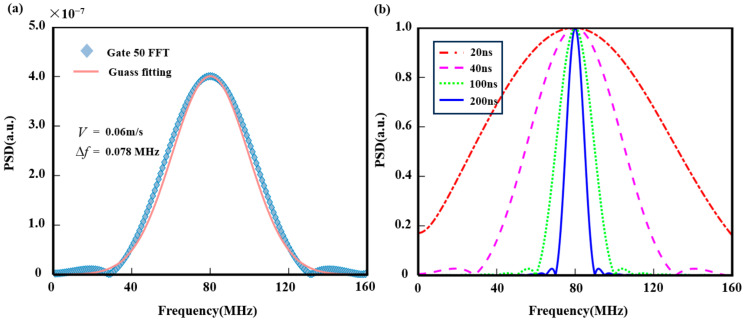
The power spectrum at 300 m. (**a**) The 40 ns pulse width power spectrum and the Gaussian fitting results; (**b**) a comparison of power spectra for four pulse widths.

First, the echo signal was windowed and transformed using the FFT to obtain the power spectrum. At a sampling rate of 1 GSa/s, the number of time-domain echo sampling points within the 1.2 km detection distance is 8000, with a range gate size of 6 m. Simulation results show that all range gates (200) within 1.2 km were transformed using FFT, with 40 sampling points per gate. During FFT, data points in each range gate were padded with zeros to 1024 points. Figure 6a shows the power spectrum and Gaussian fitting results at the 50th range gate (300 m). To verify the relationship between the spectral width and emitted laser pulse width, pulse widths of 20 ns, 40 ns, 100 ns, and 200 ns were simulated. At 300 m, the echo signal was processed with a Hanning window, and the resulting normalized power spectra for different pulse widths are shown in Figure 6b.

Gaussian fitting was used to find the spectrum’s maximum point as the carrier frequency for each range gate. In Figure 6a, the theoretical wind frequency for the 50th range gate is 0 MHz, the simulated result is 80.07 MHz, and the wind speed error is 0.06 m/s. Figure 6b shows that the power spectrum for a 20 ns pulse width is the broadest, while longer pulse widths result in progressively narrower spectra. A wider spectrum increases uncertainty in the center frequency of the Gaussian fit, thus affecting wind speed inversion accuracy.

**Figure 7:**
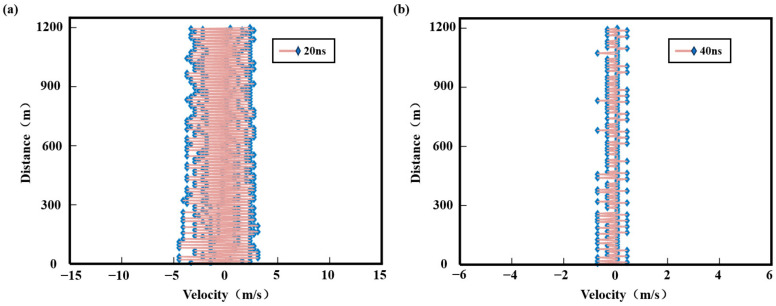
The wind speed measurement accuracy at a detection distance of 1.2 km. (**a**) The 20 ns pulse width; (**b**) the 40 ns pulse width.

Figure 7a,b displays the retrieved wind profiles within a 1.2 km range measured using 20 ns and 40 ns laser pulses, respectively. As shown in Figure 7a, the wind speed retrieval from echo signals transmitted with 20 ns laser pulses exhibits significant errors ranging from −4.3 m/s–3 m/s, rendering the measurement results unreliable. Conversely, Figure 7b demonstrates that wind speed measurements derived from 40 ns laser pulse echoes achieve errors within ±0.7 m/s. These results indicate that the 40 ns pulse width provides moderate measurement accuracy, while the 20 ns pulse width yields unsatisfactory wind speed measurements despite its higher range resolution. Therefore, enhancing wind measurement precision while maintaining high-range resolution (via narrower pulse widths like 20 ns) remains a critical research objective.

### 2.3. The Establishment of the Simulation Model for Pulse Modulation Techniques

As analyzed in the simulation results in Section 2, single-pulse energy attenuation and spectral broadening effects under short-pulse conditions significantly degrade wind speed measurement accuracy. To address this, the system incorporated an MZM-based PSK-DCP technique [15,16]. This method employs phase-modulated long–short pulse pairs for wind field detection to mitigate the adverse impacts of short pulses. The CW laser was modulated into paired probe pulses with distinct phases. Leveraging the random scattering properties of atmospheric aerosols, high-range resolution echo signals were extracted through the differential processing of the two pulses.

**Figure 8:**
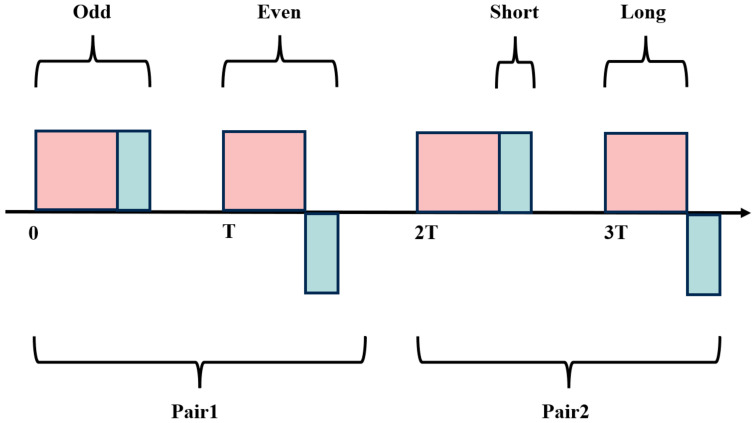
Schematic of odd–even pulse pair.

In the PSK-DCP technique, the long and short pulses are denoted as $f_{L}(t)$ and $f_{S}(t)$, respectively. The phase modulation of these pulses is critical for enhancing range resolution. A pulse pair composed of long and short pulses, both with phase +1 (corresponding to a phase shift of 0), is termed an odd pulse. When the long pulse has a phase +1 (0 phase shift) and the short pulse has a phase −1 (corresponding to a π phase shift), the pair is called an even pulse. The configurations of odd and even pulses are illustrated in Figure 8, with their mathematical representations given below:

(5) $$E_{in}^{0}(t)=E_{in}^{0}(t)e^{j(\omega t+\theta_{0})}=[f_{L}(t)+f_{S}(t)]e^{j(\omega t+\theta_{0})}$$

(6) $$\Delta E_{in}^{\pi }(t)=E_{in}^{\pi }(t)e^{j(\omega t+\theta_{\pi })}=[f_{L}(t)-f_{S}(t)]e^{j(\omega t+\theta_{\pi })}$$

where $E_{in}^{0}(t)$ and $E_{in}^{\pi }(t)$ denote the odd and even pulses, respectively; ω is the optical frequency; and $\theta_{0}$ and $\theta_{\pi }$ are the initial phases.

**Figure 9:**
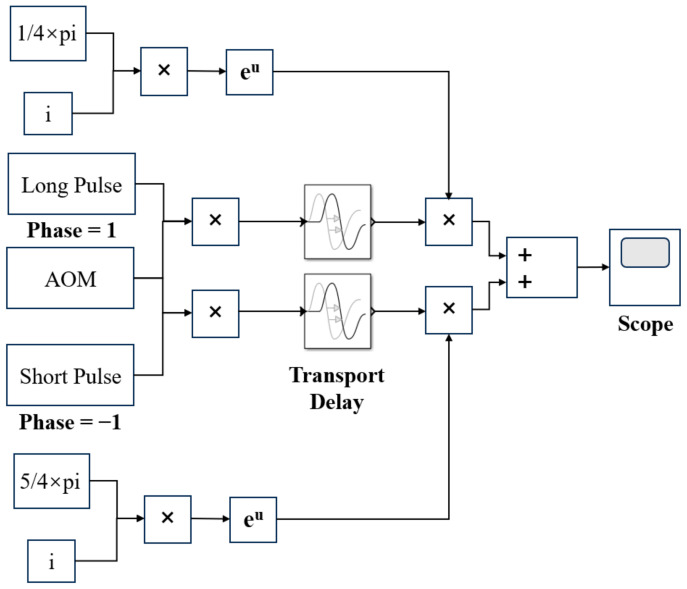
Even pulse simulation structure diagram.

In order to facilitate the modulation of odd and even pulses, the MZM module was introduced on the basis of the CDWL simulation model established in Section 2, and the simulation structure of even pulses is shown in Figure 9. First, the input to this module is the probe light modulated by the AOM. This probe light was multiplied by the long and short pulses of $f_{L}(t)$ and $f_{S}(t)$, respectively, and entered the delay module. Subsequently, the signals were multiplied with $e^{j*\frac{\pi }{4}}$ and $e^{j*\frac{5\pi }{4}}$ to construct the odd and even pulses. During the multiplication operations, it was critical to maintain consistency between the pulse widths and repetition frequency of the odd–even pulses and those modulated by the AOM simulation module.

**Figure 10:**
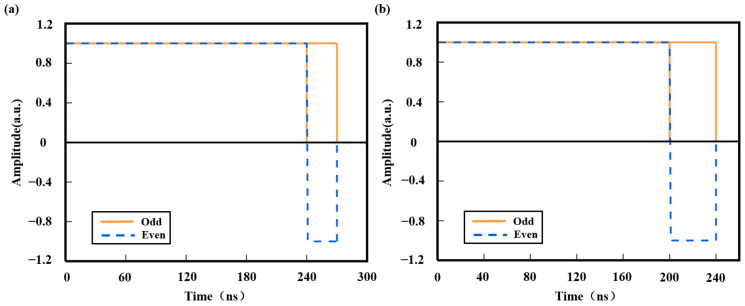
Odd–even pulse amplitude diagram. (**a**) The long and short pulse widths: 240 ns and 30 ns; (**b**) The long and short pulse widths: 200 ns and 40 ns.

The MZM module outputted odd and even pulses, as shown in Figure 10. The long and short pulse widths for the odd and even pulses in Figure 10a are 240 ns and 30 ns, respectively, while in Figure 10b, the widths are 200 ns and 40 ns, respectively. According to Figure 10b, the pulse width set by the AOM preceding the MZM module should also be at least 240 ns, enabling the intensity modulation of the pulse light while shifting the frequency.

**Figure 11:**
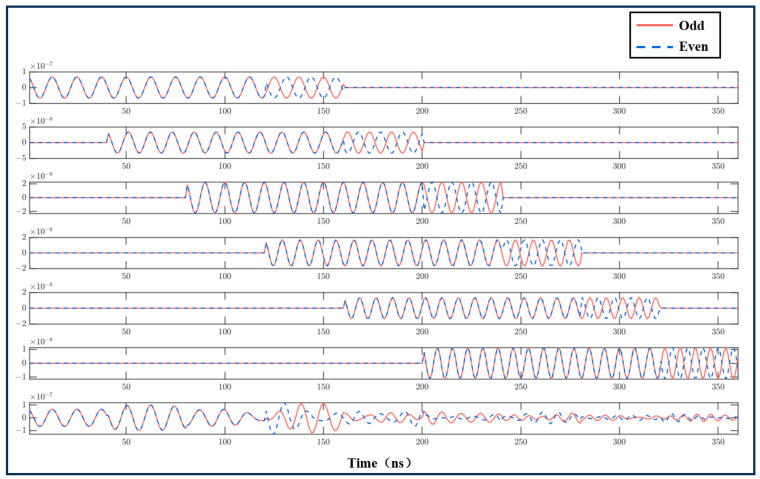
Simulation results of atmospheric stratification echo of odd–even pulse pairs.

Figure 11 presents a schematic of the simulation results for the atmospheric stratification model when transmitting odd–even pulse pairs. In the figure, red represents odd pulses, and blue represents even pulses. The ratio of long pulses to short pulses in the odd–even pulses is 3:1, with long pulses lasting 240 ns and short pulses 80 ns. The first six sub-figures depict the pulse pair echo signals for different layers, while the final sub-figure shows the superimposed result of these layered pulse pair echo signals. Figure 11 provides an intuitive understanding of the transmission characteristics of odd–even pulse signals within an atmospheric stratification framework.

In the data processing section, the differential correlation formula can be expressed as follows:

(7) $$S_{d}(\nu ,z_{0})=S_{1L}\bar{S_{1S}}-S_{2L}\bar{S_{2S}}$$

In Equation (7) $S_{iL}=FFT\{r_{L}^{\left(i\right)}(t,t_{0})\}$ and $S_{iS}=FFT\{r_{S}^{\left(i\right)}(t,t_{0})\}$. $S_{1L}$ denotes the FFT applied to the long-windowed segment of the odd-pulse echo signal and represents the complex conjugate operation. After multiple pulse-pair measurements and averaging, the estimated value of the echo signal power spectrum is given as follows:

(8) $$\mathrm{PSD}(\nu ,t_{0})=\left|\langle S_{d}(\nu ,t_{0})\rangle \right|$$

### 2.4. Simulation and Analysis of Echo Signal Under Pulse Modulation Technology

**Figure 12:**
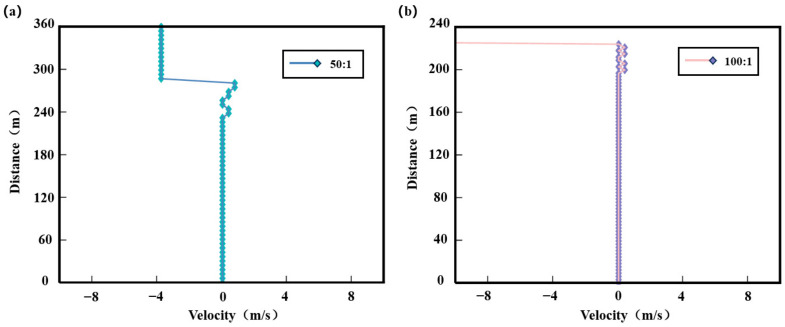
Wind field detection under different pulse width ratio modulation techniques. (**a**) Long-to-short pulse ratio of 50:1; (**b**) long-to-short pulse ratio of 100:1.

To validate whether this method can further enhance range resolution, this section modulated long–short pulse pairs and applied them to detect a simulated wind field with known parameters. The wind speed was set to constant mode with a Doppler frequency shift of 0 MHz, and the noise power was 10^−12^ W. Figure 12a shows the simulated wind profile within a 360 m detection range using a long–short pulse ratio of 50:1, where the short pulse width is 40 ns and the long pulse width is 2 μs. Figure 12b presents the wind profile within a 240 m detection range with a pulse ratio of 100:1, featuring a short pulse width of 20 ns and a long pulse width of 2 μs.

As shown in Figure 12a, with a long-to-short pulse ratio of 50:1 and a corresponding distance gate of 6 m, the wind speed measurement error within a detection distance of 240 m is 0.06 m/s. Beyond 240 m, the wind speed error increases to −4.1 m/s, significantly deviating from the theoretical wind speed. In comparison with the 20 ns short-pulse method, this mode achieves a notably reduced wind speed measurement error. In Figure 12b, the long-to-short pulse ratio of 100:1 results in a corresponding distance gate of 3 m. Within a detection distance of 200 m, the wind speed measurement error remains 0.06 m. Between 200 m and 225 m, the wind speed measurement error ranges from 0.06 m/s to 0.44 m/s. The primary source of error is the wind speed error caused by the peak frequency deviation during fitting. Beyond 225 m, the wind speed measurements significantly deviate from theoretical values, rendering the simulation results unreliable.

**Figure 13:**
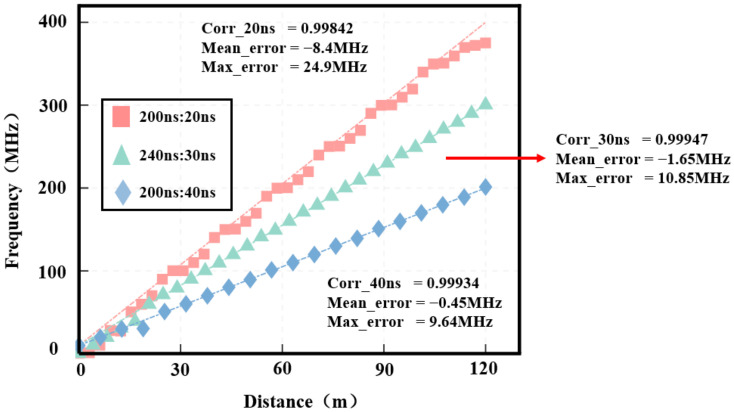
Step wind field detection with short pulses of 20 ns, 30 ns, and 40 ns.

To validate the accuracy of the pulse modulation technique in the wind lidar simulation system, the wind speed was configured as a stepped profile, with the Doppler frequency shift incrementally increasing according to the range gates corresponding to the short pulses. For instance, a 40 ns short pulse corresponds to a 6 m range gate, where the Doppler frequency shifts by 10 MHz per 6 m increment. In the pulse modulation configuration, the short pulses in the odd/even pulse pairs were set to 20 ns, 30 ns, and 40 ns, respectively. The resulting wind speed measurements are shown in Figure 13.

The red, green, and blue dashed lines in Figure 13 connect theoretical wind speeds at different range gates. Within a detection distance of 120 m, the correlation coefficient between the wind field detection results (pulses of 200 ns and 40 ns) and the theoretical wind field values is 99.934%. The average wind frequency error is −0.45 MHz, with a maximum error of 9.64 MHz at the fourth range gate. Excluding the maximum error, the average error at the remaining gates is 0.03 MHz. For pulses of 240 ns and 30 ns, the correlation coefficient between detected and theoretical values is 99.947%, with an average wind frequency error of −1.65 MHz. The wind speed error at the first five range gates is relatively high, but excluding these gates reduces the average error to 0.02 MHz, yielding accurate wind measurement results. In contrast, for pulses of 200 ns and 20 ns, the correlation coefficient is 99.842%, with an average wind frequency error of −8.40 MHz, indicating significant discrepancies between the simulation and theoretical results.

In summary, the wind measurement results with 40 ns short pulses closely align with the theoretical values. After excluding the first five range gates, the data for 30 ns short pulses are more reliable, while the results for 20 ns short pulses are deemed unreliable.

**Figure 14:**
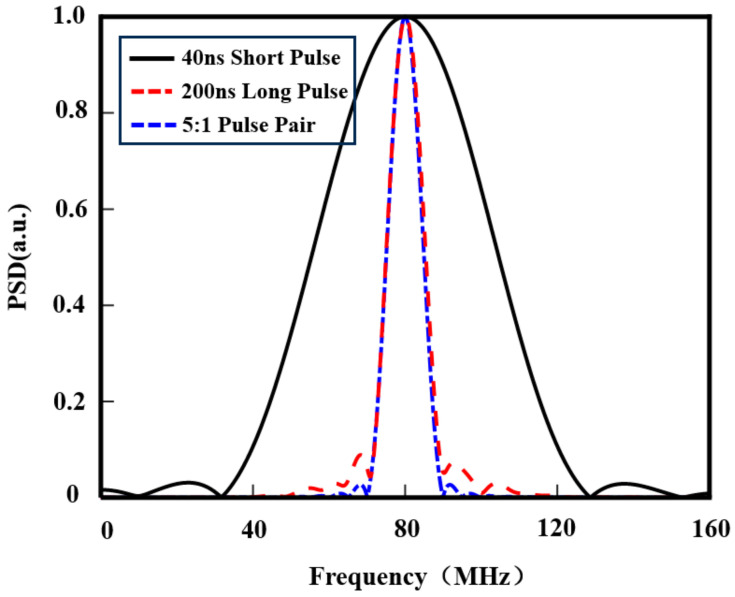
Comparison of normalized power spectra between pulse modulation techniques and long–short pulse modes.

Simulation comparisons were conducted for power spectra under pulse modulation techniques, long-pulse, and short-pulse conditions. As shown in Figure 14, the power spectrum of the pulse modulation case with a long–short pulse ratio of 5:1 nearly overlaps with that of the conventional 200 ns pulse width, whereas the short-pulse case (40 ns) exhibits a full width at half maximum (FWHM) of approximately 50 MHz. This comparative result demonstrates that the pulse modulation technique achieves narrower spectral linewidths under high-spatial-resolution conditions, which facilitates more accurate spectral centroid fitting in noisy environments, thereby reducing wind speed measurement errors.

## 3. Conclusions

This study, based on the research contexts of safe aircraft takeoff and landing in aviation safety and high-resolution wind field measurement in wind farm areas for wind power generation, focused on the range resolution enhancement mechanisms of CDWLs. An end-to-end simulation model was constructed, and a systematic simulation analysis was conducted.

First, a comprehensive simulation model of a coherent Doppler wind measurement lidar system was developed using numerical simulation tools based on the principles of CDWLs. By adjusting key parameters influencing the range resolution of wind measurements, wind speed inversion results within a detection range of 1.2 km were analyzed. The results indicate that a wind speed measurement error of ±0.7 m/s can be achieved after the lidar emits a 40 ns laser pulse and accumulates 1000 pulses. However, when the pulse width is further reduced to 20 ns, the reliability of wind speed measurements significantly decreases. Therefore, improving range resolution while maintaining wind speed measurement accuracy holds substantial research significance.

Second, the simulation system incorporated an MZM module in the hardware section and correlation algorithms in the data processing section. This system transformed conventional single-pulse detection into an odd–even pulse pair detection format, thereby enhancing range resolution through differential cross-correlation signal processing. By integrating this technique with the previously established simulation model, wind speed retrieval results from time-domain echo signals were compared with preset stepped wind speed profiles. For pulse pairs with 240 ns and 30 ns durations, the average frequency error across all range gates (excluding the first five) was 0.02 MHz, confirming the method’s validity. Furthermore, comparative analyses with short-pulse methods under varying coding schemes demonstrated that this technique maintains relatively narrow spectral widths without compromising temporal resolution, providing both simulation-based validation and theoretical foundations for practical wind field measurements.

## Figures and Tables

**Table 1:** LiDAR system simulation parameters.

		Parameter	Parameter Value
LiDAR Hardware system	CW	Continuous laser power	1 mW
		LO optical power	100 μW
	Optical Modulator	Pulse width	40 ns
		Pulse repetition frequency	10 kHz
		frequency shift	80 MHz
		Amplified pulse peak power	100 W
	Balance detection system	Balanced detector bandwidth	200 MHz
		Photoelectric conversion coefficient	0.9 A/W
Atmospheric transmission module		Total system efficiency	0.234
		Atmospheric transmittance	0.96 km^−1^
		Atmospheric backscatter coefficient	2.5 × 10^−7^ m^−1^ sr^−1^
		Telescope aperture	30 mm
		Noise power	10^−12^ W
Data processing module		Incoherent accumulation times	1000

## Data Availability

Data are contained within the article.
